# Transcriptional variation in *Babesia gibsoni* (Wuhan isolate) between in vivo and in vitro cultures in blood stage

**DOI:** 10.1186/s13071-023-05869-z

**Published:** 2023-08-07

**Authors:** Zhen Han, Yaxin Zheng, Yu Shi, Fangwei Chen, Chenglong Wu, Lingna Wang, Shiyu Lu, Dongfang Li, Xingai Guan, Lan He, Junlong Zhao

**Affiliations:** 1 State Key Laboratory of Agricultural Microbiology, College of Veterinary Medicine, Huazhong Agricultural University, Wuhan, 430070 Hubei China; 2 Key Laboratory of Preventive Veterinary Medicine in Hubei Province, The Cooperative Innovation Center for Sustainable Pig Production, Wuhan, 430070 Hubei China; 3 Key Laboratory of Development of Veterinary Diagnostic Products, Ministry of Agriculture of the People’s Republic of China, Wuhan, 430070 Hubei China

**Keywords:** *Babesia gibsoni*, Transcriptome sequencing, Asexual stage, DEGs, AP2 transcription factor, *BgAP2-M*, In vivo, In vitro

## Abstract

**Background:**

*Babesia gibsoni*, the causative agent of canine babesiosis, belongs to the phylum Apicomplexa. The development of in vitro culture technology has driven research progress in various kinds of omics studies, including transcriptomic analysis of *Plasmodium* spp. between in vitro and in vivo environments, which has prompted the observation of diagnostic antigens and vaccine development. Nevertheless, no information on *Babesia* spp. could be obtained in this respect, which greatly hinders the further understanding of parasite growth and development in the blood stage.

**Methods:**

In this study, considerable changes in the morphology and infectivity of continuous in vitro cultured *B. gibsoni* (Wuhan isolate) were observed compared to in vivo parasites. Based on these changes, *B. gibsoni* (Wuhan isolate) was collected from both in vivo and in vitro cultures, followed by total RNA extraction and Illumina transcriptome sequencing. The acquired differentially expressed genes (DEGs) were validated using qRT-PCR, and then functionally annotated through several databases. The gene with the greatest upregulation after in vitro culture was cloned from the genome of *B. gibsoni* (Wuhan isolate) and characterized by western blotting and indirect immunofluorescence assay for detecting the native form and cellular localization.

**Results:**

Through laboratory cultivation, multiple forms of parasites were observed, and the infectivity of in vitro cultured parasites in dogs was found to be lower. Based on these changes, Illumina transcriptome sequencing was conducted, showing that 377 unigenes were upregulated and 334 unigenes were downregulated. Notably, an AP2 transcription factor family, essential for all developmental stages of parasites, was screened, and the transcriptional changes in these family members were tested. Thus, the novel AP2 transcription factor gene (*BgAP2-M*) with the highest upregulated expression after in vitro adaptation was selected. This gene comprises an open reading frame (ORF) of 1989 base pairs encoding a full-length protein of 662 amino acids. *BgAP2-M* contains one AP2 domain and one ACDC conserved domain, which may be involved in the nuclear biology of parasites. The prepared polyclonal antibodies against the *BgAP2-M* peptides further detected a native size of ~ 73 kDa and were localized to the nuclei of *B. gibsoni*.

**Conclusion:**

This study presents a thorough transcriptome analysis of *B. gibsoni* in vivo and in vitro for the first time, contributing to a detailed understanding of the effects of environmental changes on the growth and development of parasites in the blood stage. Moreover, it also provides a deeper investigation for the different members of the ApiAP2 transcription factor family as various life stage regulators in *Babesia* spp.

**Graphical Abstract:**

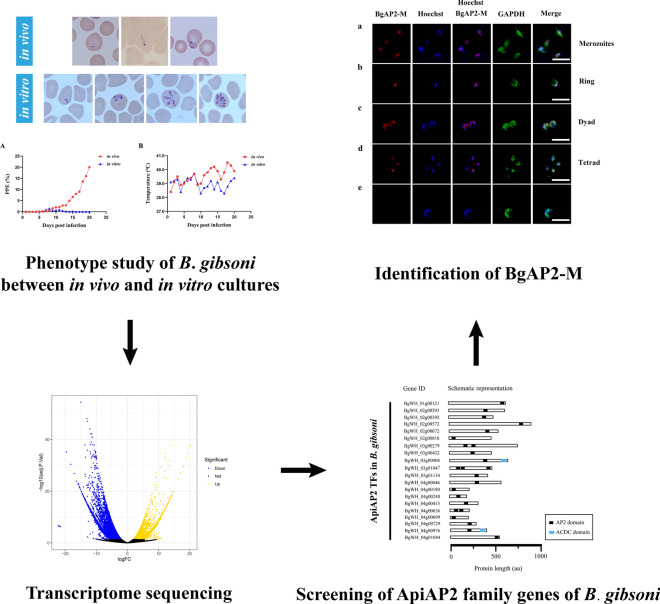

**Supplementary Information:**

The online version contains supplementary material available at 10.1186/s13071-023-05869-z.

## Background

*Babesia* species are obligate intraerythrocytic hemoprotozoa that are taxonomically classified in the phylum Apicomplexa, class Piroplasmea, order Piroplasmida, and family Babasidae [1–3]. *Babesia gibsoni* is a hemoprotozoan parasite that causes typical canine babesiosis symptoms such as hemolytic anemia, hemoglobinuria, hypotensive shock, and death [4–6]. This organism is also transovarially transmitted by *Haemaphysalis longicornis*, which is widely distributed in hilly wildland [7–10].

Laboratory in vitro culture systems have been established for various apicomplexan parasites including *Babesia bovis*, *B. bigemina*, *B. gibsoni*, *B. orientalis*, *Plasmodium falciparum,* and *P. knowlesi* [11–15]. Due to several in vitro environmental factors, including physical, nutritional, and immunological factors, that are distinct from those in vivo, parasites tend to show visible and meaningful changes in morphology, infectivity, and virulence. For instance, in *B. gibsoni* Oita isolate, long-term in vitro cultivation resulted in larger and multi-formed parasites in host erythrocytes and lower parasite infectivity in dogs [13, 16]. Hence, the deeper transcriptional changes are thought to be provoking and significant. However, such changes in genes affected by in vitro culture have been investigated in both *P. falciparum* and *P. knowlesi* [17–20]. Such alterations occasionally signify parasites' adaptation to the surrounding environment, producing better survival and propagation [21]. For example, some sexual-stage genes (gamete antigen 27/25) and others related to asexual-stage invasion and growth (MSP7, DOC2, and CLAMP) have been identified with relatively large transcription variations because they are far less likely to be transmitted to new mosquito vectors and more likely to invade host erythrocytes in in vitro environments [17, 19]. Notably, various ApiAP2 transcription factors also underwent some degree of transcriptional change. This protein family, with one to three DNA-binding domains, was determined to be the largest transcription factor family in apicomplexan organisms and precisely regulates all developmental stages of parasites [22, 23]. In *P. falciparum*, two AP2 transcription factors, PF3D7_1222600 (PfAP2-G) and PF3D7_1222400 (PfAP2-G4), were well documented to have loss-of-function nonsense mutations with premature stop codons, leading to tremendous repression of gene transcription during gametocytogenesis. Another AP2 transcription factor (PF3D7_1342900, PfAP2-HS) was detected with three nonsense mutations, all of which resided upstream of the predicted AP2 domains and truncated the full-length protein. These transcription-affected mutations in PfAP2-HS likely resulted from improved tolerance to temperature fluctuations in in vitro environments [18]. Moreover, recent studies have observed increased transcript levels of an AP2 gene of unknown function (PF3D7_0420300), which may be involved in asexual reproduction [19].

Since the establishment of in vitro culture in apicomplexan species several decades ago, the understanding of parasite biology has greatly accelerated, and a few breakthroughs have been made in vaccine development and drug target identification. In *Plasmodium* spp., transcriptomic studies have been conducted on parasites from in vitro and in vivo environments and have identified meaningful DEGs that may affect multiple biological processes. However, little information is available on *Babesia* spp. Here, we first performed transcriptome sequencing of *B. gibsoni* (Wuhan isolate) in vivo and in vitro cultures in the blood stage based on changes in parasite morphology, infectivity, and virulence. Thus, genes with significantly differential expression at the transcript level were identified.

Furthermore, the ApiAP2 transcription factor family was analyzed by quantitative real-time polymerase chain reaction (qRT-PCR), leading to the observation of a novel AP2 gene (*BgAP2-M*) with the greatest upregulation after in vitro culture. The expression and localization of *BgAP2-M* were studied by western blotting and indirect immunofluorescence assay (IFA), revealing its important role in the blood stage. Altogether, our research provides comprehensive insight into the transcriptional variation in *B. gibsoni* (Wuhan isolate) transitioning from in vivo to in vitro environments and will greatly assist in screening candidate antigens for future diagnostic usage.

## Methods

### Experimental animals

Four female beagles (1 year old) were purchased from the Anlu Laboratory Animal Center and confirmed to be free of natural *Babesia* by microscopic examination of Giemsa-stained blood smears and PCR. The animals were provided standard amounts of food and drinking water daily. Two beagles were used as erythrocyte and serum donors. After splenectomy, two other experimental beagles were inoculated with 2 × 10^8^ erythrocytes infected with *B. gibsoni* (Wuhan isolate) in vivo and in vitro. Rectal temperature was recorded daily, and blood samples were collected daily to monitor parasitemia. Once it exceeded 20%, venous blood samples were collected for further analysis.

### In vitro culture of *B. gibsoni* blood stage

According to previously established protocols (unpublished) in the laboratory, the culture medium was RPMI 1640 (Gibco) containing 0.9 mM sodium pyruvate, 24 mM NaHCO_3_ with a pH of 6.9, penicillin G at 200 units/ml, streptomycin at 200 μg/ml, and including 20% dog serum for in vitro culture. Normal dog erythrocytes were supplied with EDTA-K_2_ anticoagulant from donor dogs. The whole blood was washed three times with Roswell Park Memorial Institute (RPMI) 1640 medium by centrifugation at 500×*g* for 10 min at 4 °C, and the leukocyte layer was removed. The washed dog erythrocytes were added to a twofold volume of RPMI 1640 medium and stored at 4 °C for further use. Normal dog serum was collected from donor dogs and stored at −20 °C until use. In a 24-well plastic culture plate, 100 μl erythrocytes were suspended in 900 μl culture medium to reach a final packed cell volume (PCV) of 10%. Every 24 h, approximately 800 μl of culture supernatant was removed without disturbance of sedimented erythrocytes, and replaced with refreshing medium. Microscopic examination of the sedimented erythrocytes was conducted daily to monitor parasitemia. Subcultures were prepared every to 3–4 days. When the parasitemia reached 8%, a 100-μl mixture of culture medium, which contained approximately 8 × 10^6^ infected erythrocytes, was transferred into a new well containing suspension of 800 μl fresh culture medium and 100 μl normal dog erythrocytes. The parasites were cultivated in the incubator containing 5% CO_2_ at 37 °C. *Babesia gibsoni* (the Wuhan isolate) was stably cultivated for 150 passages.

### Enrichment of blood-stage *B. gibsoni* both in vivo and in vitro

Sedimented *B. gibsoni* (Wuhan isolate)-infected erythrocytes in vivo (P0) and in vitro (P150, 150 generations) were collected. Briefly, the blood samples were centrifuged at 500×*g* for 10 min at 4 °C. Then a 10-fold volume of red blood cell (RBC) lysis buffer (Biosharp, Hefei, China) was added to the collected erythrocytes, followed by incubation at 4 °C for 15 min. The lysates were filtered through a 3-μm membrane (Whatman, Florham Park, NJ, USA) and centrifuged at 10,000×*g* at 4 °C for 15 min to obtain the enriched parasite pellets.

### RNA extraction

The collected parasite pellets were resuspended in 1 ml TRIzol^®^ reagent (TransGen Biotech, Beijing, China) and stored at −80 °C until use. To extract RNA, 200 μl of chloroform per ml of TRIzol was added to sample supernatants, followed by a vortex of 30 s and incubation at 4 °C for 5 min. Samples were centrifuged at 10,000×*g* at 4 °C for 15 min to separate phases. The upper aqueous phase was transferred to a new microfuge tube containing an equal volume of isopropyl alcohol, followed by a static incubation for 10 min. For isolating total RNA, samples were centrifuged at 10,000×*g* at 4 °C for 15 min, and the supernatant was removed. The enriched RNA pellets were mixed with 1 ml 75% ethanol and centrifuged at 10,000×*g* at 4 °C for 15 min. After RNA pellets were dissolved in 30 μl diethyl pyrocarbonate (DEPC)-treated water, quality control was conducted using a NanoDrop 2000 spectrophotometer (Thermo Fisher Scientific) and Agilent 2100 Bioanalyzer (Agilent Technologies).

### RNA-Seq library preparation and sequencing

RNA sequencing (RNA-Seq) library construction was performed using Novogene. Poly(A)+ RNA (messenger RNA [mRNA]) was enriched using magnetic beads with oligo (DT) and fragmented using a fragmentation buffer. After single-stranded complementary DNA (cDNA) synthesis with random hexamers, buffer, dNTPs, DNA polymerase I, and RNase H were used to synthesize double-stranded (ds) cDNA. The acquired ds cDNA was purified with AMPure XP beads (Beckman Coulter, Brea, CA, USA), followed by end-repaired, 3′ ends adenylated, and barcoded adaptor ligated to the end. Fragments were enriched by PCR to obtain the final libraries, followed by primary quantification using Qubit^®^ 2.0 dsDNA High Sensitivity Assay Kit (Thermo Fisher Scientific). The diluted libraries were accurately assayed using qRT-PCR and sized using an Agilent 2100 Bioanalyzer (Agilent Technologies). The multiplexed RNA-Seq libraries were sequenced using 150-base-pair (bp) paired-end reads on a NovaSeq 6000 (Illumina) platform.

### Assembly and gene function annotation

Clean data in the FASTQ format were obtained by removing reads containing an adaptor, poly-N, and low-quality reads (< Q20) using Cutadapt (version 1.15). Subsequently, the clean data's effective rate, error rate, Q20, Q30, and guanine–cytosine (GC) content were calculated and validated. High-quality clean reads were assembled using Trinity (version 2.12.0) [24] with K-mer = 25 bp to obtain the assembled transcripts and unigenes. The parameters of the *B. gibsoni* genome (unpublished) were set in the Trinity software to acquire cleaner unigene sequences without host transcripts. Gene function annotation was based on several online public databases, including the National Center for Biotechnology Information (NCBI) non-redundant protein sequences (NR), Kyoto Encyclopedia of Genes and Genomes (KEGG), Gene Ontology (GO), eggNOG (evolutionary genealogy of genes: Non-supervised Orthologous Groups), Swiss-Prot, and Pfam databases.

### Expression and DEG analysis

The assembled unigene sequences were mapped with clean reads using RSEM (recursive structural equation model) software (default parameters) [25] to estimate gene and isoform expression levels. DEGs were analyzed using DESeq2 software (version 1.32.0). The criteria of *P*-adjusted value < 0.05 [26] and |log2FoldChange|> 1 were set to screen significant DEGs. TransDecoder software (version 5.5.0) was used to identify the candidate coding regions within the transcript sequences. EggNOG-mapper v2 (http://eggnog-mapper.embl.de/) was used online for functional annotation and domain prediction of candidate open reading frames (ORF), including GO term annotation and KEGG pathway enrichment.

### qRT-PCR assays

Several genes were randomly selected to confirm the accuracy of the analyzed transcriptome data using qRT-PCR. RNA was isolated by the same method as described previously. TURBO DNase (Thermo Fisher Scientific, USA) was used to thoroughly remove DNA contamination remaining in the extracted RNA samples. The concentration and absorbance ratios for 260/280 nm and 260/230 nm of post-treatment RNA were determined using a NanoDrop 2000 spectrophotometer (Thermo Fisher Scientific). A quantity of 400–500 ng of total RNA from each parasite preparation was reverse-transcribed into cDNA using a PrimeScript RT Reagent Kit with gDNA (genomic DNA) Eraser (TaKaRa Bio). qRT-PCR was carried out using the ChamQ Universal SYBR qPCR Master Mix (Vazyme, Nanjing, China) on a QuantStudio^®^5 qRT-PCR system (Applied Biosystems, USA). Specific primers were designed using Clone Manager software. Glyceraldehyde-3-phosphate dehydrogenase (GAPDH) was the reference gene for normalizing target gene expression. The 2^−ΔΔCt^ method was used for calculating the relative expression level of unigenes [27].

### Gene amplification and bioinformatics analysis

The *BgAP2-M* gene was amplified from genomic DNA and cDNA using specific primers. The amino acid (aa) sequence of *BgAP2-M* was translated using ORF Finder (https://www.ncbi.nlm.nih.gov/orffinder). The Simple Modular Architecture Research Tool (SMART) was used to predict the conserved domains in the *BgAP2-M* aa sequence [28]. The nuclear localization signal (NLS) was predicted using NLStradamus [29]. Multiple protein sequence alignments were performed using ClustalW [30]. MEGA7 software was used for the phylogenetic analysis of *BgAP2-M* among homologs in other apicomplexan species, namely *B. bovis*, *B. bigemina*, *B. divergens*, *B. ovata*, *B. microti*, *Theileria annulata*, *P. falciparum*, *P. yoelii*, and *P. berghei* [31]. SWISS-MODEL was used for tertiary structure homology modeling of the AP2 domain of *BgAP2-M* [32].

### Synthetic peptides and polyclonal antibodies

Peptides ranging from 12 to 15 aa were synthesized based on the protein sequence of *BgAP2-M* as follows: MPDKRQRRSSRKKLS (288–302 aa) and KQYTSNLTDEQKSHR (531–545 aa). Two New Zealand white rabbits were immunized with peptides conjugated to keyhole limpet hemocyanin (KLH), followed by six booster inoculations at 2-week intervals. Immune serum titers were assessed by enzyme-linked immunosorbent assay (ELISA), and the rabbits were exsanguinated 10 days after the last inoculation. Purified polyclonal antibodies were used for subsequent immunoblotting and IFA.

### Immunoblot assays

The saponin-lysed total parasite protein for immunoblot was prepared from *B. gibsoni *in vivo and in vitro blood samples, then separated on 12.5% sodium dodecyl sulfate–polyacrylamide gel electrophoresis (SDS-PAGE), and transferred to polyvinylidene fluoride (PVDF) membranes. The membranes were blocked with 5% (w/v) skim milk in Tris-buffered saline Tween 20 (TBST) for 2 h at room temperature and then probed with primary antibodies against *BgAP2-M* and histone H3 (Proteintech, USA) at 4 °C overnight. After three washes in TBST, the membranes were incubated with the appropriate horseradish peroxidase (HRP)-conjugated secondary antibody for 1 h at room temperature and detected using enhanced chemiluminescence reagents (Advansta, USA).

### Indirect immunofluorescence assays

IFAs were performed to detect the localization of *BgAP2-M*. The in vitro cultured *B. gibsoni* smears were air-dried and fixed with cold 95% methanol and 5% acetone (v/v) at −20 °C for 20 min. Prepared samples were permeabilized with 0.05% Triton X-100 in phosphate-buffered saline (PBS) for 10 min and then washed three times with PBS. The slides were incubated with primary antibodies against *BgAP2-M* and GAPDH (Proteintech, USA) diluted 1:1000 in 3% bovine serum albumin (BSA)/PBS at 4 °C overnight, followed by incubation with the secondary antibodies Alexa Fluor 594-conjugated goat anti-rabbit immunoglobulin G (IgG) and Alexa Fluor 488-conjugated goat anti-mouse IgG (Thermo Fisher Scientific, USA) diluted 1:1000 in 3% BSA/PBS at 37 °C for 1 h. Nuclear staining of the parasites was performed using Hoechst 33342 at room temperature for 10 min. Images were acquired using a fluorescence microscope.

## Results

### Morphological observation of continuous in vitro cultured *B. gibsoni* (Wuhan isolate)

**Fig. 1:**
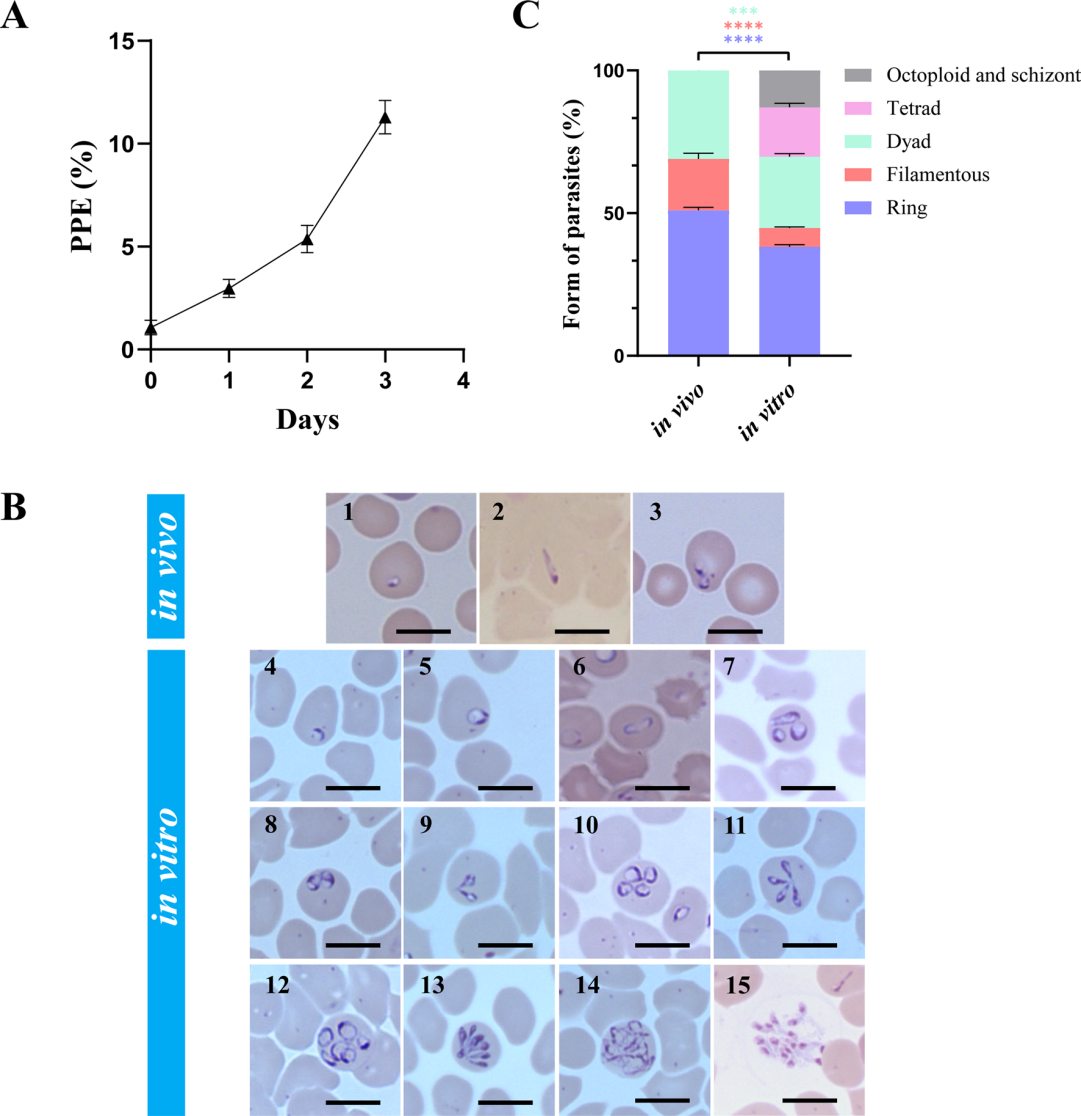
Changes in parasitemia and morphology of in vitro cultured *B. gibsoni* (Wuhan isolate). **A** Changes in parasitemia of in vitro cultured *B. gibsoni* (Wuhan isolate) within 3 days after *splitting*. **B** Morphological changes in *B. gibsoni* (Wuhan isolate) after in vitro adaptation. (1–3) The small dot-form, filamentous, and dyad parasites of in vivo environment. (4–7) Early rings, mature rings, filamentous and multiple parasites of in vitro environment. (8–13) Young dyad, mature dyad, young tetrads, mature tetrads, young octoploid, mature octoploid parasites of in vitro environment. (14, 15) Schizont and merozoites egressing from erythrocytes. Bars, 5 μm. **C** The proportion of different forms of parasites from in vivo and in vitro environments containing 10% *B. gibsoni*-infected erythrocytes. The statistical analysis: ****P < 0.0001, ***P < 0.001, **P < 0.01, *P < 0.05, ns: not significant (multiple Student’s *t*-tests). The error bars represent mean ± standard deviation (SD) for three biological replicates

*Babesia gibsoni* was successfully cultured in vitro in 20% serum. After splitting, parasitemia reached 10% ± 1.5% on day 3 (Fig. 1A).

Morphological observations of *B. gibsoni* (Wuhan isolate) in the vertebrate host showed small ring *and* dot forms upon microscopic inspection of Giemsa-stained cells (Fig. 1B). The small ring-form parasites were 1.75 ± 0.26 μm × 1.85 ± 0.30 μm in mean size. Neither extracellular nor multiple forms of parasites, such as tetrads or octoploids, were observed [13]. However, after continuous in vitro cultivation, different forms of parasites were observed, including larger ring-form parasites, filamentous-form parasites, dyad-form parasites, tetrad-form parasites, even octoploid parasites, and schizonts (Fig. 1B). When parasitemia increased significantly, many egressing merozoites were observed outside the host erythrocytes, and two different forms of parasites grew in one erythrocyte. On day 3 of the 150th generation, with a parasitemia of 10%, erythrocytes parasitized with the ring-form parasites had the largest proportion of all infected erythrocytes. In contrast, octoploid and schizont-parasitized erythrocytes had the lowest proportions (Fig. 1C).

### Infectivity examination of in vivo and in vitro cultured *B. gibsoni* (Wuhan isolate)

**Fig. 2:**
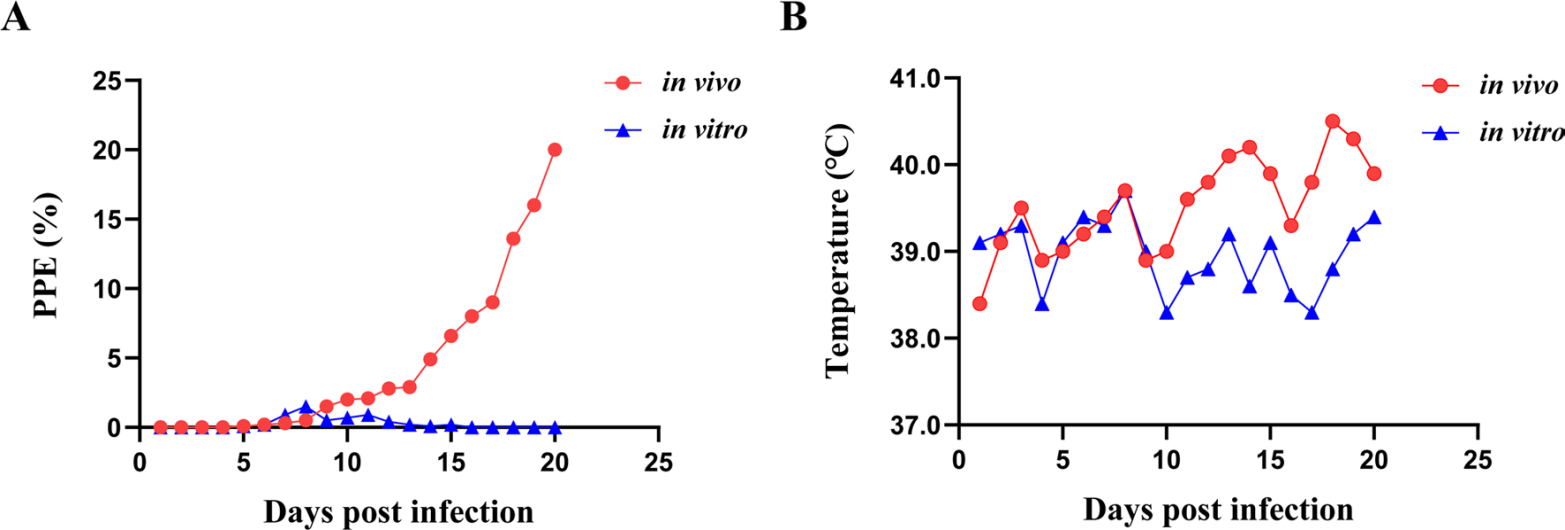
Changes in parasitemia and temperature after infection with 2 × 10^8^ infected erythrocytes from *B. gibsoni* (Wuhan isolate) in vivo and in vitro cultures. **A** Changes in parasitemia of two dogs infected with *B. gibsoni* (Wuhan isolate) from in vivo (red) and in vitro cultures (blue), respectively. **B** Changes in the temperature of two dogs infected with *B. gibsoni* (Wuhan isolate) from in vivo (red) and in vitro cultures (blue), respectively

The infectivity of the dogs was examined by inoculating 2 × 10^8^ infected erythrocytes intravenously from in vivo blood samples or in vitro cultures. Changes in parasitemia and temperature over time after inoculation are shown in Fig. 2. The parasitemia of a dog infected with *B. gibsoni* (Wuhan isolate) in vivo parasites slowly increased to 3% during the first 10 days. At the same time, the body temperature did not fluctuate strongly and remained in the range of 38.5–39.5 °C. However, the parasitemia increased significantly the following week and reached 21% by day 20, accompanied by obvious hyperthermia. On the contrary, a dog infected with continuous in vitro cultured *B. gibsoni* (Wuhan isolate) did not show a high level of parasitemia. However, the peak of parasitemia was not more than 2%. The body temperature remained normal and did not exceed 40 °C during the experiment.

During in vitro adaptation, environmental variations significantly influence parasite blood-stage development, such as morphological changes or infectivity of natural hosts. To further identify DEGs based on these variations, we generated RNA-Seq data on blood-stage *B. gibsoni* (Wuhan isolate) from in vivo and in vitro cultures.

### De novo transcriptome sequencing, assembly, and DEG function annotation

Qualified cDNA libraries were prepared from *B. gibsoni* (Wuhan isolate) in vivo and in vitro cultured parasites with three biological replicates. Raw reads were from 24.47 to 30.36 Gb after Illumina sequencing and deposited in the NCBI Sequence Read Archive (SRA) database. After removing unqualified and low-quality reads, clean reads were obtained with a range from 23.55 to 29.16 Gb and used for the following analysis. Using Trinity software and *B. gibsoni* (Wuhan isolate) genome guidance, 9561 transcripts, and 1340 unigenes were assembled for subsequent analysis of DEGs.

**Fig. 3:**
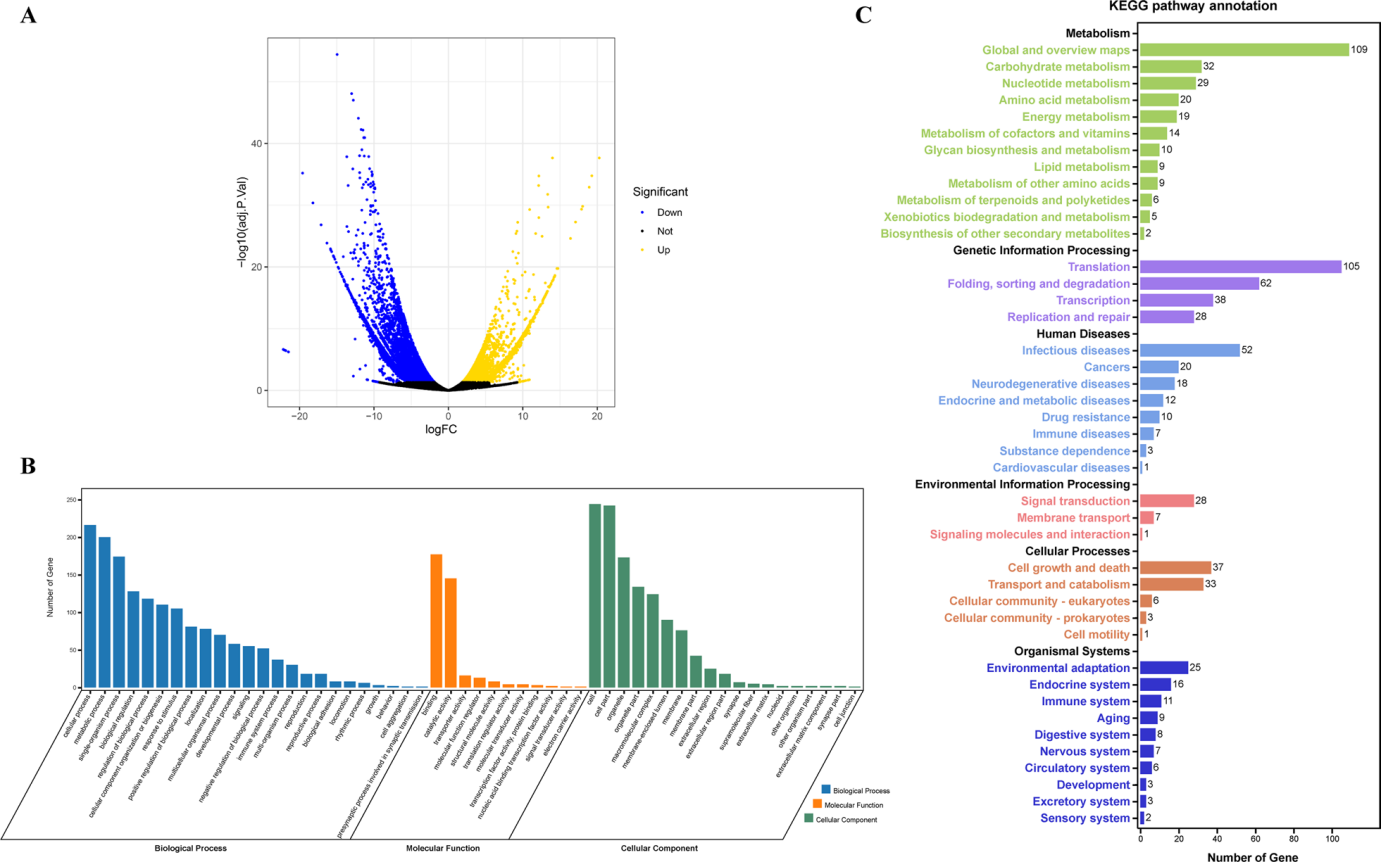
GO term annotation and KEGG pathway enrichment of significant DEGs between in vitro and in vivo groups. **A** Volcano plot of relative mRNA expression from *B. gibsoni* (Wuhan isolate) continuous in vitro cultured parasites compared to in vivo original parasites. Genes at left (blue) and right (yellow) are downregulated and upregulated DEGs, respectively. **B** GO term annotation of significant DEGs. **C** KEGG pathway enrichment of screened significant DEGs

DEGs were screened to identify differences between the experimental and control groups. Based on a set of criteria of *P*-adjusted value < 0.05 and |log2FoldChange|> 1, 711 unigenes were identified as significant DEGs when comparing in vitro to in vivo groups. A total of 377 unigenes were upregulated, and 334 unigenes were downregulated (Fig. 3A). To better understand the function of the significant DEGs, open reading frames (ORF) were predicted using TransDecoder software and subjected to the eggNOG-mapper online service for further GO analysis and KEGG pathway enrichment (Fig. 3B, C) (Additional file 1: Table S1). For GO functional annotation, unigenes were classified into three main GO categories: biological processes, molecular functions, and cellular components. Most unigenes were assigned to cellular and metabolic processes within the biological process category. More than 150 unigenes were assigned catalytic activity and binding terms for molecular functions. Among the last category, cellular components, terms named cell and cell parts had a larger proportion of unigenes than any other term. For KEGG pathway enrichment, all the unigenes were assigned to six main categories: metabolism, genetic information processing, human diseases, environmental information processing, cellular processes, and organismal systems. Among these, metabolism, translation, signal transduction, cell growth, and environmental adaptation pathways were the main enrichment factors that might be related to the parasite’s asexual-stage development during in vitro adaptation.

**Fig. 4:**
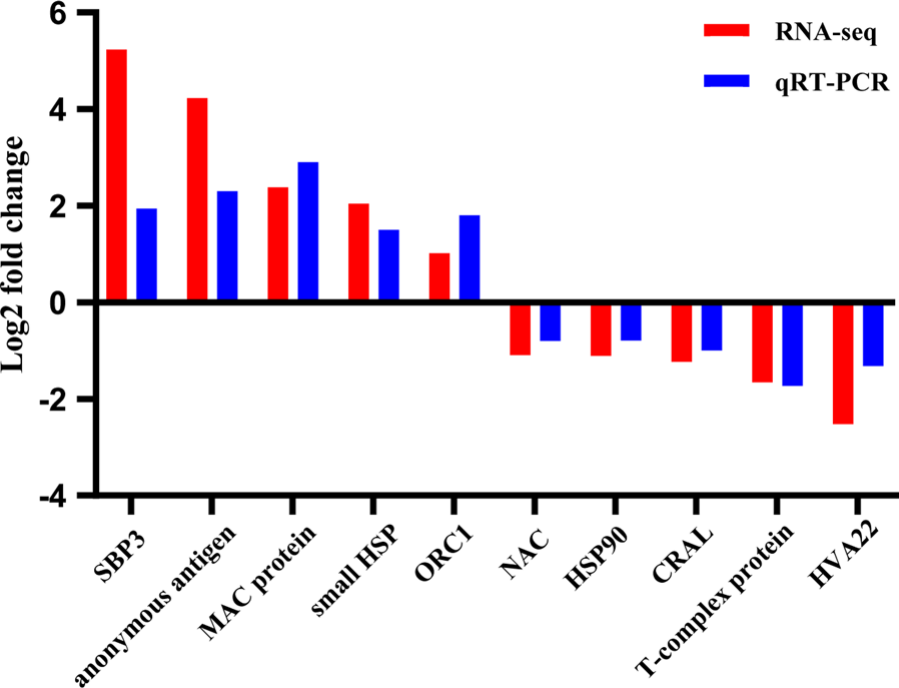
RNA-Seq data validation from *B. gibsoni* (Wuhan isolate) in vivo and in vitro cultured groups. qRT-PCR validation of expression level of randomly selected DEGs. Expression levels were normalized to GAPDH and presented relative to controls

To verify the accuracy of the RNA-Seq data, qRT-PCR was performed using 10 randomly selected unigenes (Fig. 4). The results of qRT-PCR demonstrated similar expression patterns to the RNA-Seq data, even though there were differences in the log2 fold change between RNA-Seq and qRT-PCR data due to differences between methods. The specific primer sequences are listed in Additional file 2: Table S2.

### Identification of genes with significant differential expression

Considerable changes were found in the transcript levels of genes involved in parasite invasion of red blood cells, parasite virulence, and asexual- and sexual-stage development (Table 1). We found two essential genes encoding merozoite surface protein 2 and microneme protein 14 that had higher transcript levels in *B. gibsoni* (Wuhan isolate) in vitro culture groups. These two genes may be involved in the host red blood cell invasion process, consistent with the observable phenomena of rapid asexual proliferation and high parasitemia. Moreover, data analysis revealed that the transcript levels of a gene encoding spherical body protein 3 increased markedly during in vitro cultivation. Previous studies showed that spherical body protein 2 truncated copy 11(*sbp2t11)* of *B. bovis* was identified as a specific attenuation marker because of its consistent pattern of upregulation in four geographically distinct attenuated strains compared to their virulent parental strains [33]. However, spherical body protein 3 of *B. gibsoni* (Wuhan isolate) had low identity with spherical body protein 2 truncated copy 11 of *B. bovis*, so whether SBP3 of *B. gibsoni* (Wuhan isolate) is related to loss or gain of virulence needs to be thoroughly investigated. Of the DEGs in the RNA-Seq data, two genes encoding hook-associated protein 2 (hap2) and the AP2 transcription factor (AP2-G) were downregulated after continuous in vitro culturing. In *P. falciparum*, two ApiAP2 transcription factor genes (PF3D7_1222600 and PF3D7_1222400) involved in gametocytogenesis were previously identified in convergent nonsense mutants after long-term laboratory in vitro adaptation, resulting in more asexual stages of the parasite in vitro growth [18]. Similarly, the decreased expression of sexual-stage-related genes, including hook-associated protein 2 (hap2) and AP2 transcription factor (AP2-G), after continuous in vitro adaptation may contribute to asexual-stage survival and growth. Conversely, vertebrate hosts provide gametocytes with a more natural environment conducive to tick transmission; therefore, these sexual-stage genes would have a higher expression level under these conditions.

**Table 1:** Unigenes showing significantly different transcription levels in *B. gibsoni* (Wuhan isolate) blood-stage development between in vivo and in vitro cultured groups

DEGs	Transcript ID	Product annotation	Log2 fold change
Upregulated DEGs	TRINITY_GG_16_c41_g1	Merozoite surface protein 2	7.70
	TRINITY_GG_7_c11_g2	Spherical body protein 3	4.23
	TRINITY_GG_2_c55_g2	Microneme protein14	2.32
	TRINITY_GG_3_c127_g1	AP2 domain transcription factor	2.06
	TRINITY_GG_2_c139_g2	Calcium-dependent protein kinase 4	1.70
	TRINITY_GG_3_c159_g2	AP2 domain transcription factor	1.26
Downregulated DEGs	TRINITY_GG_2_c226_g2	Thioredoxin family protein, putative	−3.73
	TRINITY_GG_2_c163_g2	Cyclin-dependent kinase binding protein, putative	−3.65
	TRINITY_GG_16_c43_g1	T-complex protein 1	−2.51
	TRINITY_GG_2_c61_g5	AP2 domain transcription factor	−1.92
	TRINITY_GG_3_c199_g1	TPR domain-containing protein	−1.65
	TRINITY_GG_3_c219_g1	Hook-associated protein 2	−1.45
	TRINITY_GG_2_c74_g1	Zinc finger protein, putative	−1.25
	TRINITY_GG_16_c69_g2	AP2 domain transcription factor	−1.22

### Screening of the AP2 transcription factor family in *B. gibsoni* (Wuhan isolate)

**Fig. 5:**
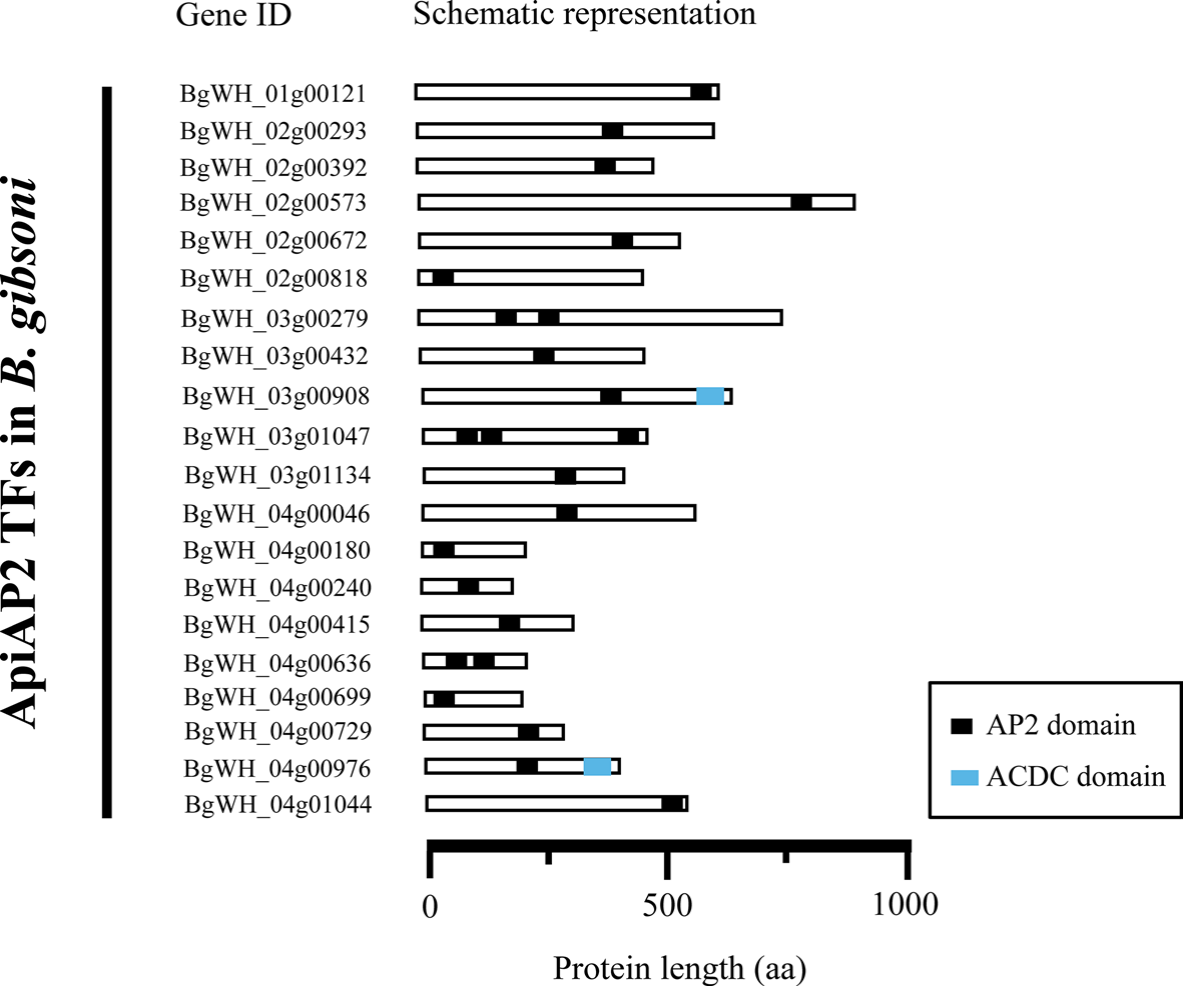
Schematic representation of the location and number of AP2 and ACDC domains in the AP2 transcription factor family of *B. gibsoni* (Wuhan isolate). AP2 and ACDC domains are shown in black and blue, respectively. The gene ID and protein length of every AP2 transcription factor of *B. gibsoni* (Wuhan isolate) are also indicated

ApiAP2 transcription factors have been functionally identified in malarial and *Toxoplasma* parasites. This transcription factor family can bind to genomic DNA via sequence-specific motifs in the promoter and regulate every developmental stage of apicomplexan parasites. In our RNA-Seq data analysis, multiple AP2 transcription factor genes were upregulated or downregulated after continuous in vitro adaptation, revealing significant transcriptome changes in the entire AP2 gene family during *B. gibsoni* (Wuhan isolate) blood-stage development. We present a systematic screen of the AP2 gene family in the *B. gibsoni* (Wuhan isolate) genome (unpublished data). Some AP2 genes were significantly differentially expressed, while others were obtained using tBLASTn with homologous species, such as *B. bovis* or *B. microti.* Thus, 20 genes encoding AP2 domain-containing proteins were present in *B. gibsoni* (Wuhan isolate) (Fig. 5).

The aa sequences of *B. gibsoni* (Wuhan isolate) AP2 proteins were predicted using ORF Finder (https://www.ncbi.nlm.nih.gov/orffinder). The predicted protein lengths were highly diverse, ranging from 184 to 924 aa. Additionally, conserved functional domains were bioinformatically predicted using the SMART tool, showing different AP2 domains distributed across AP2 transcription factors. Most of these predicted AP2 proteins contained a single AP2 domain, whereas three members were identified with more than one AP2 domain, such as two or three. We suggest that different AP2 domains of the same protein bind to distinct DNA motifs and sequentially regulate the expression of diverse target genes. A similar regulatory mechanism was previously identified in *P. falciparum*, including PfAP2-I (PF3D7_1007700) [34]. The AP2 transcription factor contains three AP2 domains that can recognize multiple distinct DNA sequences; however, only the third domain is essential for blood-stage development. Two AP2 proteins with an ACDC domain (an AP2-coincident domain, mostly at the C-terminus) were detected. This conserved domain (approximately 80–90 aa) is mainly located at the C-terminus of the AP2 transcription factor and resides in other apicomplexan parasite AP2 proteins, including *B. bovis*, *P. falciparum*, and *T. gondii* [35]*.* However, the involvement of this uncharacterized domain in nuclear biology requires further investigation. Furthermore, neither transmembrane regions nor signal peptides were identified in any *B. gibsoni* (Wuhan isolate) AP2 proteins.

**Fig. 6:**
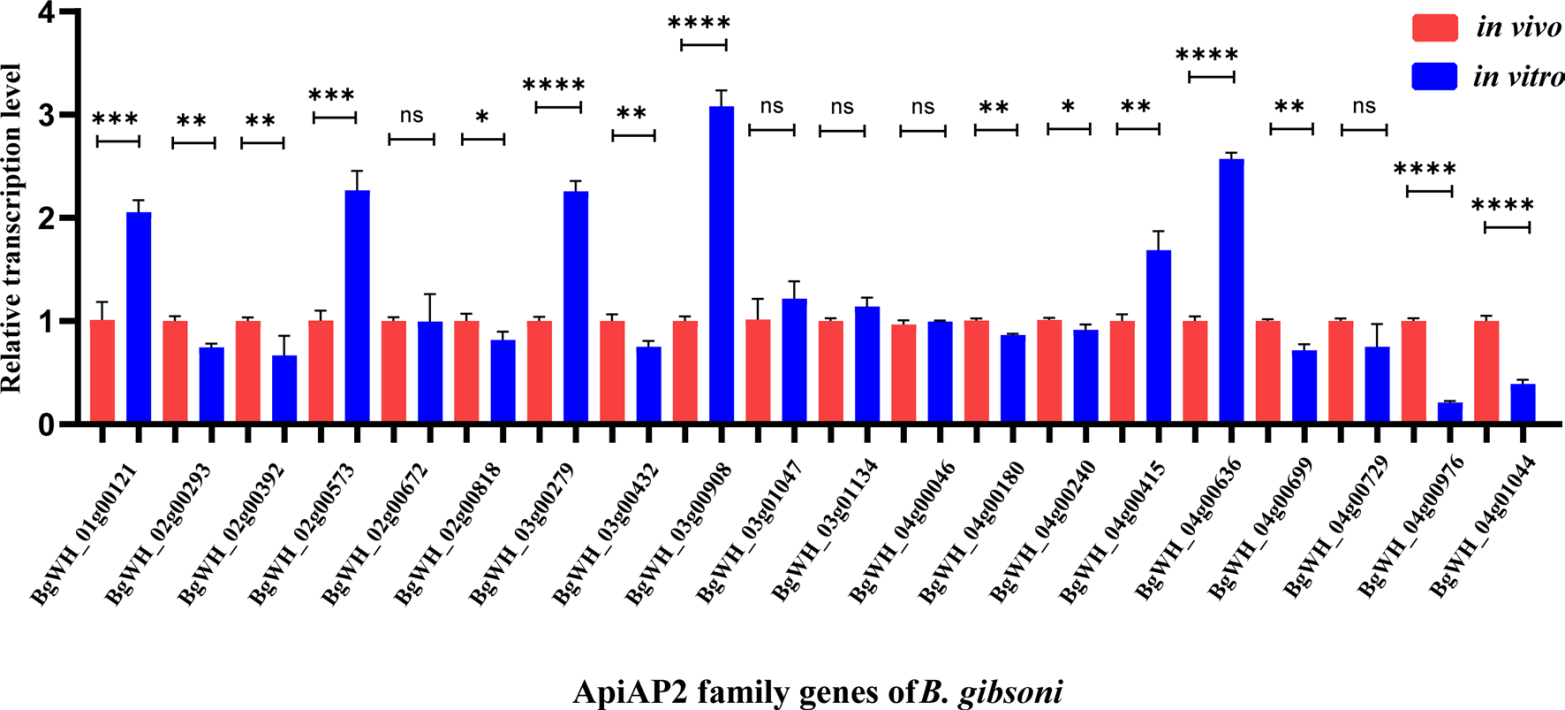
Relative transcription level of AP2 family genes of *B. gibsoni* (Wuhan isolate) between in vivo (red) and in vitro cultured (blue) blood-stage parasites by qRT-PCR assays. The statistical analysis: ****P < 0.0001, ***P < 0.001, **P < 0.01, *P < 0.05, ns: not significant (unpaired two-tailed Student’s *t*-test). The error bars represent mean ± SD for three biological replicates

Given that this transcription factor family serves as a key regulator of parasite development, we performed qRT-PCR to analyze the transcription level changes in the entire AP2 gene family (Fig. 6). Primer sequences used for this assay are listed in Additional file 3: Table S3. Two AP2 genes (BgWH_04g01044 and BgWH_04g00976) were significantly downregulated. It has been proposed that these two AP2 proteins may regulate the parasite's sexual-stage development, similar to AP2-G [36]. Subsequently, we focused on several upregulated AP2 genes, including BgWH_03g00908 (unknown function), BgWH_04g00415 (unknown function), BgWH_03g00279 (homolog of PfSIP2), BgWH_04g00636 (unknown function), BgWH_01g00121 (homolog of PfAP2-O), and BgWH_02g00573 (unknown function). In *P. falciparum*, PfSIP2 was previously described as a heterochromatin-associated transcription factor, which binds exclusively to subtelomeric regions and silences the transcription of the downstream *var* gene family [37]. Likewise, PfAP2-O was recently shown to influence the transcriptional memory of *var* genes in asexual stages and contribute to functional midgut-invading ookinetes at the mosquito stage [38–40]. The homolog of PfAP2-O in *Toxoplasma gondii* called TgAP2XI-5, was also demonstrated to drive the transcription of crucial virulence factor genes [41]. Thus, BgWH_03g00279 and BgWH_01g00121 may play important roles in regulating the expression of genes related to virulence or parasite proliferation. Interestingly, one AP2 gene (BgWH_03g00908) was found to have threefold upregulated transcription levels after in vitro cultivation; however, homologs of BgWH_03g00908 in other apicomplexan parasites, such as *P. falciparum* (PF3D7_1239200) and *B. bovis* (BBOV_IV011830), remain largely unknown. Its significantly increased expression after in vitro adaptation is likely to illustrate potential roles for regulating parasite merogony during the asexual stage of development. Hence, this AP2 transcription factor was selected for further exploration and designated *BgAP2-M*.

### Characterization of BgAP2-M of *B. gibsoni* (Wuhan isolate)

We first cloned this gene from gDNA and cDNA of *B. gibsoni* (Wuhan isolate). The *BgAP2-M* gene contained a 1989-bp ORF with no introns, encoding a predicted protein of 73 kDa.

**Fig. 7:**
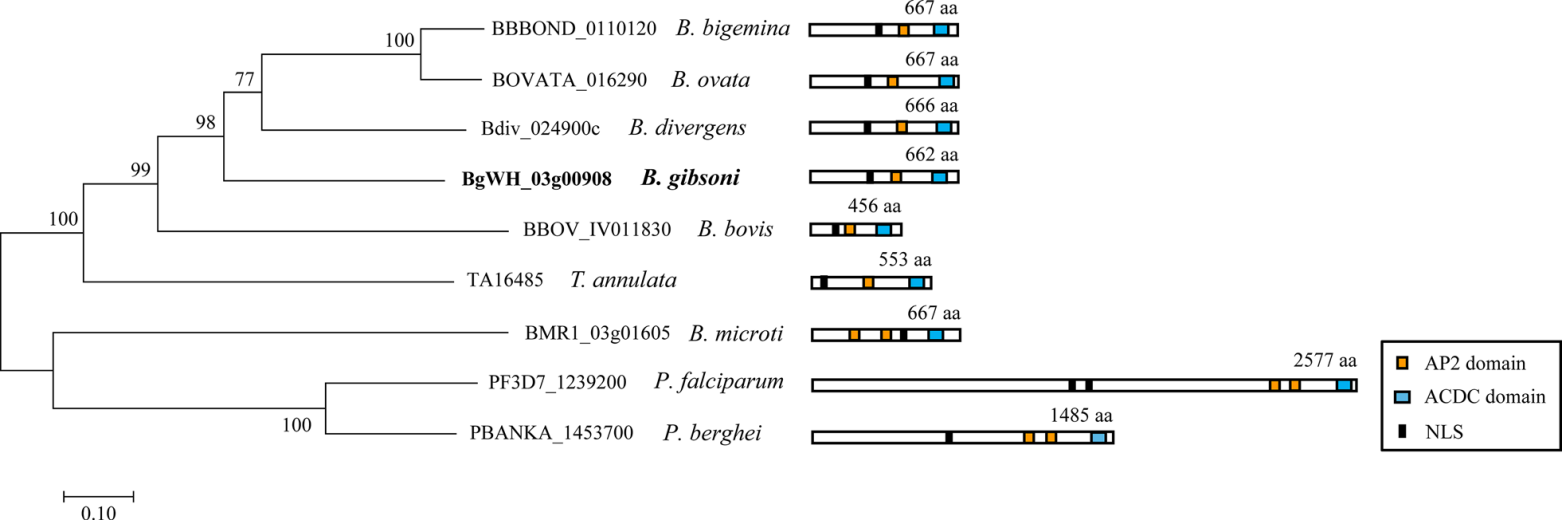
Phylogenetic analysis and schematic diagrams of *BgAP2-M* among apicomplexan parasites. Left panel shows phylogenetic relationship of the *BgAP2-M* protein sequences containing AP2 conserved domains with homologs of other apicomplexan parasites using the neighbor-joining method. The tree is drawn to scale, with branch lengths measured in the number of substitutions per site. The accession numbers of related proteins are marked and obtained from PiroplasmaDB and PlasmoDB online databases. Right panel shows schematic diagrams of *BgAP2-M* and related proteins in other apicomplexan parasites. Conserved domains including AP2 domains (orange) and ACDC domains (blue) are highlighted by different rectangles, as well as nuclear localization signals (NLS)

The full-length *BgAP2-M* protein was aligned with putative orthologs of other apicomplexan species, including *B. bigemina*, *B. ovata*, *B. divergens*, *B. bovis*, *B. microti*, *T. annulata*, *P. falciparum*, and *P. berghei,* to perform the phylogenetic analysis (Fig. 7). *BgAP2-M* was grouped into one clade with Bdiv_024900c, BOVATA_016290, and BBBOND_0110120, revealing a close relationship. BBOV_IV011830 appeared to have a relatively lower identity with BgAP2-M than with the orthologs of *B. bigemina*, *B. ovata*, and *B. divergens.* Similar cases have been reported for other AP2 transcription factor family members in *B. gibsoni* (Wuhan isolate). Unsurprisingly, PF3D7_1239200 and PBANKA_1453700 claded with each other and were distantly related to *BgAP2-M*, probably because of their different taxonomic order. In addition, schematic diagrams of *BgAP2-M* and the related proteins are presented. *BgAP2-M* and its orthologs in *B. bigemina*, *B. ovata*, *B. divergens*, *B. bovis* and *T. annulata* possess a single AP2 domain and an ACDC domain. In contrast, BMR1_03g01605, PF3D7_1239200, and PBANKA_1453700 had two AP2 domains distributed at different protein positions, indicating the binding of distinct DNA sequences. As functional transcription factors imported into the nuclear environment, classical NLS were predicted using NLStradamus online tools. The prediction identified a 20-amino-acid length of NLS in *BgAP2-M* and related proteins of other apicomplexan parasites, implying potential NLS-mediated nuclear import.

**Fig. 8:**
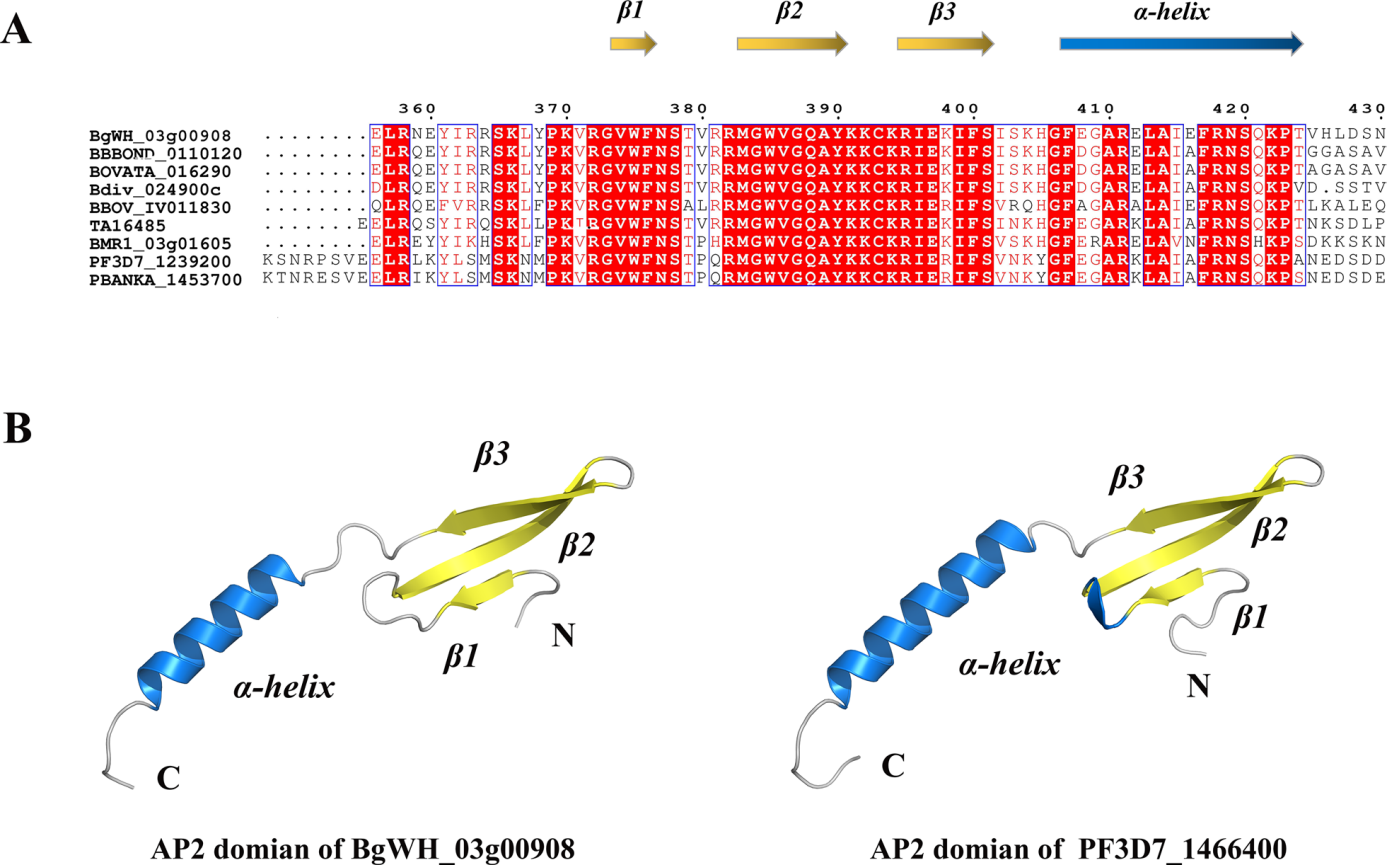
Multiple alignments and tertiary structure prediction of AP2 conserved domain in *BgAP2-M*. **A** AP2 domains of *BgAP2-M* and similar orthologues of other apicomplexan parasites were compared with accession numbers marked on the left of the diagram. The predicted secondary structures were highlighted with arrows of different colors, β-strands (yellow) and α-helix (blue). **B** The tertiary structure prediction of *BgAP2-M* AP2 domain. Left panel: predicted tertiary structure of AP2 domain of *BgAP2-M* (generated using SWISS-MODEL). Right panel: the published tertiary structure of PF3D7_1466400's AP2 domain. The β-strands (yellow) and α-helix (blue) were depicted

Moreover, the AP2 domain of *BgAP2-M* was well conserved among other apicomplexan parasites, as shown in Fig. 8A. Extraordinary sequence similarities suggested the presence of identical DNA motifs in the genome. TA13515 (orthologue of PF3D7_1222600 PfAP2-G) has been previously found to bind to identical GxGTACxC motifs [42]. The highly similar region has a classical secondary structure, three strands and one helix, indicating an analogous way to bind DNA. Through tertiary structure homology modeling using PF3D7_1466400 as a template [43], the three-strand and one-helix canonical structures were predicted, as shown in Fig. 8B.

### Expression and localization of *BgAP2-M* of *B. gibsoni* (Wuhan isolate)

**Fig. 9:**
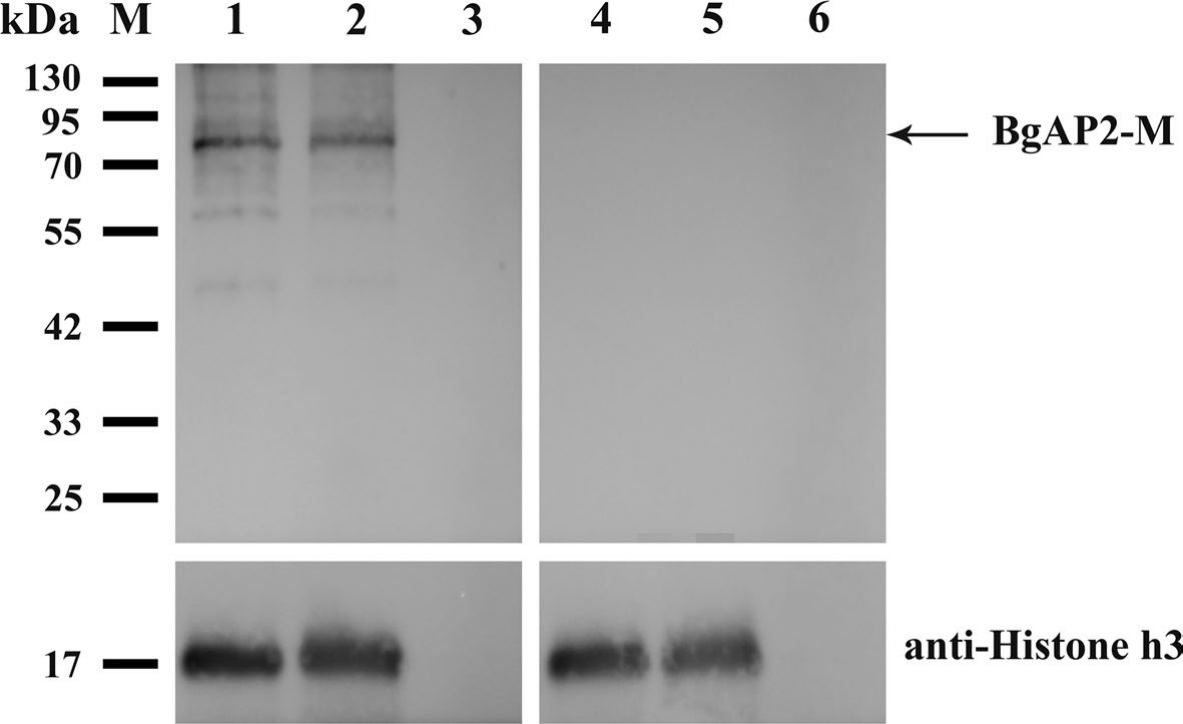
Immunoblotting of native *BgAP2-M* protein from in vitro cultured or in vivo* B. gibsoni* (Wuhan isolate). Lane M: molecular weight marker; Lane 1: lysate of in vitro cultured *B. gibsoni* probed with rabbit polyclonal antibodies against BgAP2-M peptides; Lane 2: lysate of in vivo* B. gibsoni* probed with rabbit polyclonal antibodies against *BgAP2-M* peptides; Lane 3: lysate of noninfected *Canis* erythrocytes probed with rabbit polyclonal antibodies against *BgAP2-M* peptides; Lane 4: lysate of in vitro cultured *B. gibsoni* probed with pre-immune rabbit serum; Lane 5: lysate of in vivo* B. gibsoni* probed with pre-immune rabbit serum; Lane 6: lysate of noninfected *Canis* erythrocytes probed with pre-immune rabbit serum. Arrows indicate the corresponding bands. Anti-histone H3 antibody was used as a loading control to detect the expression of *BgAP2-M*

Prepared polyclonal antibodies against *BgAP2-M* peptides were used to identify the expression of the native *BgAP2-M* protein in *B. gibsoni* (Wuhan isolate). A specific band of approximately 73 kDa was detected in the lysate of in vitro cultured or in vivo* B. gibsoni* (Wuhan isolate), consistent with the predicted molecular weight of the mature protein (Fig. 9). In contrast, no products were detected in the negative control group. Detection of histone H3 protein demonstrated equal protein loading across different lanes.

**Fig. 10:**
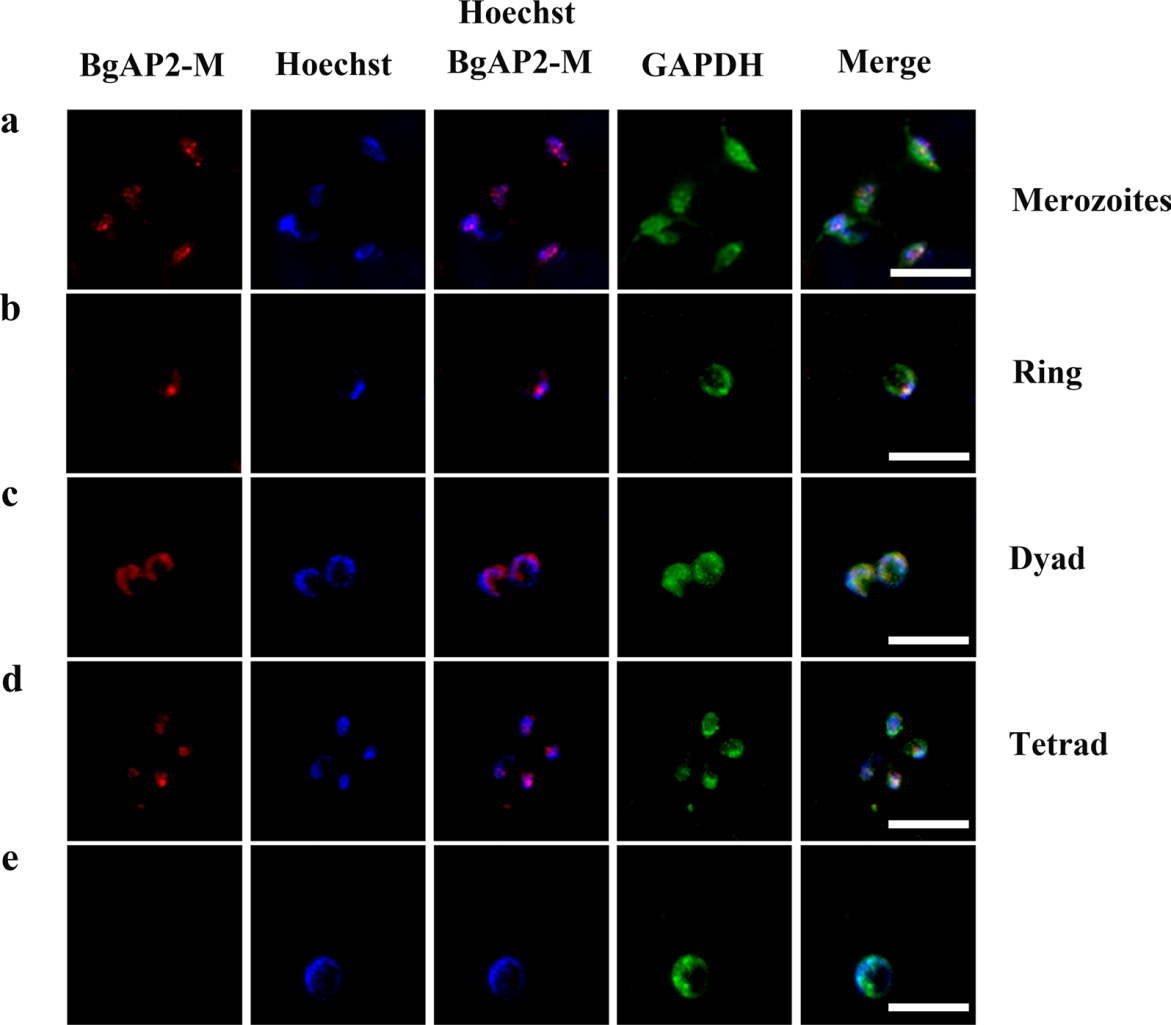
Indirect immunofluorescence assays detecting the location of native *BgAP2-M* protein in *B. gibsoni* (Wuhan isolate). The *BgAP2-M* protein and the parasite nuclei were stained with polyclonal antibodies against *BgAP2-M* peptides (red) and Hoechst 33,342 (blue), respectively. Anti-GAPDH antibody was used to stain the cytosol of parasites (green). **a** Extracellular merozoites. **b** Ring stage. **c** Dyad stage. **d** Tetrad stage. **e** Negative control: the primary antibody was pre-immune rabbit serum. Scale bar = 5 μm

The localization of *BgAP2-M* was determined by an IFA using polyclonal antibodies against *BgAP2-M* peptides. The IFA showed strong reactivity with the antiserum in both the intra- and extra-erythrocytic parasites of *B. gibsoni* (Fig. 10). As expected, *BgAP2-M* was localized to the nuclei of *B. gibsoni*. However, no fluorescence signal was observed in the negative control group. The cytosol of *B. gibsoni* was stained with antibodies against GAPDH.

## Discussion

In this study, based on the successful establishment of an in vitro culture system of *B. gibsoni* (Wuhan isolate), the changes in morphology and infectivity of *B. gibsoni* (Wuhan isolate) were observed and identified after long-term in vitro cultivation. Transcriptome sequencing was performed using in vivo and in vitro cultured *B. gibsoni* (Wuhan isolate) at the blood stage. Some DEGs related to the growth, invasion, and virulence of parasites were identified, and an ApiAP2 transcription factor family was identified, including a novel AP2 transcription factor (*BgAP2-M*), which may be involved in the merogony of parasites in the asexual stage. The native *BgAP2-M* protein had a molecular weight of approximately 73 kDa, consistent with the predicted size. Furthermore, the *BgAP2-M* protein was localized in the nucleus of *B. gibsoni,* including the intra- and extra-erythrocytic stages, which shared a common phenomenon with other AP2 transcription factors identified in *Plasmodium* spp. and *Toxoplasma* spp.

In vitro culture systems of several *Babesia* species have been established, including *B. bovis*, *B. bigemina*, *B. gibsoni*, *B. duncani*, and *B. orientalis*, amongst others [11–13, 44, 45], providing a convenient platform for genetic manipulation and research on the interaction between parasites and hosts. However, during continuous in vitro cultivation, the morphology and infectivity of parasites seem to undergo considerable changes, perhaps due to differences in nutrient levels or physical environmental factors [21]. For example, in a previous study, *B. gibsoni* Oita isolate was successfully cultivated in vitro for 738 days in the 214th subculture; however, the size of the parasites increased, and multiple forms of parasites were observed, such as large oval or petaloid-formed parasites. Simultaneously, the infectivity and virulence of parasites were strongly decreased in natural hosts [13, 16]. Similarly, *B. gibsoni* (Wuhan isolate) underwent greater morphological changes from small ring-form parasites to large ring-form parasites, dyad-form parasites, tetrad-form parasites, octoploid parasites, and schizonts. In addition, the infectivity in dogs was observed to be lower.

To further investigate the transcriptional variation in genes based on these changes in the morphology and infectivity of parasites after in vitro adaptation, transcriptome sequencing was carried out on blood-stage *B. gibsoni* (Wuhan isolate) from in vivo and in vitro cultured groups. Notably, some genes involved in the parasite invasion process demonstrated increased expression after in vitro adaptation, such as merozoite surface protein 2 and microneme protein 14; this presumably resulted from increased asexual propagation and high parasitemia in the in vitro environment, which was free from the pressure of the host immune system. Similarly, in *P. falciparum*, claudin-like apicomplexan microneme protein (CLAMP) as an invasion factor was also shown to have a higher transcript level in the long-term in vitro-adapted strains than in the clinical in vivo isolates [19]. Additionally, the transcript level of spherical body protein 3 in *B. gibsoni* (Wuhan isolate) was also remarkably increased. Early studies on *B. bovis* determined that spherical body protein 2 truncated copy 11(*sbp2t11)* plays an important role as an attenuation marker when comparing four distinct attenuated strains to their corresponding virulent strains [33].

Nevertheless, SBP3 of *B. gibsoni* (Wuhan isolate) shared low similarity with *sbp2t11* of *B. bovis* even though both belong to the spherical body protein family. Therefore, whether the upregulation of the SBP3 gene represents the lower virulence of *B. gibsoni* (Wuhan isolate) requires further experimental confirmation. For *Plasmodium* spp. and *Babesia* spp., laboratory in vitro-adapted strains usually lack access to transmission vectors (mosquitoes or ticks); thus, they experience a more asexual erythrocytic stage, as well as a less sexual stage. Consequently, genes coding for sexual-stage antigens were downregulated in the in vitro cultured parasites compared with the in vivo isolates. For example, sexual-stage-related genes (gamete antigen 27/25) and the AP2 transcription factor gene (AP2-G), which are essential to be downregulated in both *P. falciparum* and *P. knowlesi* laboratory-adapted strains, which were essential for sexual-stage commitment and development [17]. As to *B. gibsoni* (Wuhan isolate), it was no exception that similar sexual-stage proteins (hap2 and AP2-G) were found to be downregulated to prevent gametocytogenesis.

Interestingly, several AP2 transcription factors were upregulated or downregulated, and a similar phenomenon has been observed in other *Plasmodium* species. In 2005, the ApiAP2 (apicomplexan AP2) transcription factor family was first described as containing conserved domains similar to the Apetala2/ethylene response factor (AP2/ERF) DNA-binding domain in plants [22, 46], and so far is well identified in *Plasmodium* spp. or *Toxoplasma* spp. Previous studies on *P. falciparum* culture adaptation in the laboratory have revealed transcriptional changes in multiple AP2 transcription factor genes, including ApiAP2 genes (PF3D7_1222600, PF3D7_1222400, and PF3D7_1342900), which were revealed to have loss-of-function mutations with premature stop codons [18]. These adaptive nonsense mutations could likely produce proportionally more asexual stages for survival and propagation, together with progressive endurance of frequent temperature fluctuations during in vitro cultivation. We showed transcriptional changes in all AP2 transcription factor members of *B. gibsoni* (Wuhan isolate) using qRT-PCR assays. Notably, two AP2 transcription factor genes (BgWH_04g01044, homolog of BBOV_III009600, and BgWH_04g00976, homolog of BBOV_III008870) were downregulated during in vitro adaptation. It was hypothesized that these two transcription factors might regulate sexual-stage development. Therefore, gene disruption and chromatin immunoprecipitation (ChIP)-seq assays are urgently needed in future studies. More striking was the prominent upregulation of five AP2 transcription factor genes, two of which are homologs of PfAP2-O and PfSIP2. Genome-wide ChIP assays described high-affinity binding with promoters of subtelomeric virulence gene families for PfAP2-O and PfSIP2, significantly influencing the parasite asexual-stage growth [37, 39]. The upregulation of PfAP2-O and PfSIP2 homologs in *B. gibsoni* (Wuhan isolate) was likely associated with increased asexual-stage cycles and propagation caused by culture adaptation. Intriguingly, one AP2 transcription factor of *B. gibsoni* (Wuhan isolate) (a homolog of PF3D7_1239200 or BBOV_IV011830) was upregulated after in vitro cultivation, indicating its considerable role in regulating the transcription of genes related to merogony during the asexual stage. We identified a novel AP2 transcription factor, *BgAP2-M*. *BgAP2-M* was revealed to have only one AP2 domain through multiple alignments, which showed high aa sequence similarity with other apicomplexan species.

Moreover, a canonical secondary structure of three strands and one helix was predicted, suggesting a more stable recruitment upstream of the target genes. Furthermore, a novel ACDC (AP2-coincident domain, mostly at the C-terminus (ACDC), was detected and is well conserved across apicomplexan parasites. Notably, this unknown domain is exclusively located at the C-terminus of AP2 transcription factors. Therefore, the co-occurrence of these two domains may imply potential roles in gene transcription, chromatin modification, and remodeling [35]. To further characterize the native *BgAP2-M* protein, polyclonal antibodies against *BgAP2-M* peptides were prepared. The size of native *BgAP2-M* was ~ 73 kDa in the lysate of in vitro cultured or in vivo *B. gibsoni* (Wuhan isolate), as determined by western blotting, which was consistent with the theoretical prediction. IFA revealed the localization of the nuclei of *B. gibsoni*. Intense fluorescence signals were observed in either extracellular egressing merozoites or intracellular parasites of the ring-, dyad-, and tetrad-form stages, implying a possible role for *BgAP2-M* in merogony development. Future studies should focus on functional experiments, such as constructing gene disruption mutants or performing ChIP-seq assays. However, the homologs of *BgAP2-M* in other *Plasmodium* species (*P. falciparum*, *P. berghei*, and *P. yoelii*) were unsuccessful to be directly disrupted at the blood stage after several attempts, indicating its extremely important role in parasite development [47–49].

## Conclusion

Transcriptome sequencing was performed for the first time on *B. gibsoni* (Wuhan isolate) in vivo and in vitro cultures based on changes in morphology and infectivity. From this, a series of DEGs were discovered, allowing a more sophisticated understanding of pathways related to parasite invasion, virulence, and asexual- and sexual-stage growth. Further qRT-PCR analysis of the AP2 transcription factor family of *B. gibsoni* (Wuhan isolate) identified a novel AP2 transcription factor (*BgAP2-M*) with a size of ~ 73 kDa. It localized in the nuclei of *B. gibsoni* from extracellular and intracellular stages. The present study thoroughly explains transcriptional changes in *B. gibsoni* (Wuhan isolate) after continuous in vitro cultivation. It reports a novel AP2 transcription factor (*BgAP2-M*) that may regulate the merogony of parasites during the asexual stage of development. However, the specific target genes regulated by BgAP2-M in the asexual stage remain unknown, and further research is warranted.

### Supplementary Information

**Additional file 1: Table S1.** GO terms and KEGG pathway annotations of DEGs in this study.

**Additional file 2: Table S2.** qRT-PCR primers used to validate RNA sequencing data.

**Additional file 3: Table S3.** qRT-PCR primers for the ApiAP2 family genes of *B. gibsoni.*

## Data Availability

All RNA-Seq data used in this study have been deposited in the NCBI SRA database (accession numbers: SRR24288283, SRR24288284, SRR24288285, SRR24288286, SRR24288287, and SRR24288288).

## Abbreviations

gDNA: Genomic DNA
cDNA: Complementary DNA
qRT-PCR: Quantitative real-time PCR
RSEM: Recursive structural equation model
BLAST: Basic Local Alignment Search Tool
KLH: Keyhole limpet hemocyanin
SDS-PAGE: Sodium dodecyl sulfate–polyacrylamide gel electrophoresis
TBST: Tris-buffered saline with Tween 20
PBS: Phosphate-buffered saline
HRP: Horseradish peroxidase
NLS: Nuclear localization signal
BSA: Bovine serum albumin
